# Illuminating Music: Impact of Color Hue for Background Lighting on Emotional Arousal in Piano Performance Videos

**DOI:** 10.3389/fpsyg.2022.828699

**Published:** 2022-03-15

**Authors:** James McDonald, Sergio Canazza, Anthony Chmiel, Giovanni De Poli, Ellouise Houbert, Maddalena Murari, Antonio Rodà, Emery Schubert, J. Diana Zhang

**Affiliations:** ^1^Empirical Musicology Laboratory, School of the Arts and Media, University of New South Wales, Sydney, NSW, Australia; ^2^Centro di Sonologia Computazionale, Department of Information Engineering, University of Padova, Padua, Italy; ^3^The MARCS Institute for Brain, Behavior and Development, Western Sydney University, Sydney, NSW, Australia; ^4^Independent Researcher, Sydney, NSW, Australia; ^5^School of Chemistry, University of New South Wales, Sydney, NSW, Australia

**Keywords:** color, music, performance, lighting, emotion, arousal, red, blue

## Abstract

This study sought to determine if hues overlayed on a video recording of a piano performance would systematically influence perception of its emotional arousal level. The hues were artificially added to a series of four short video excerpts of different performances using video editing software. Over two experiments 106 participants were sorted into 4 conditions, with each viewing different combinations of musical excerpts (two excerpts with nominally high arousal and two excerpts with nominally low arousal) and hue (red or blue) combinations. Participants rated the emotional arousal depicted by each excerpt. Results indicated that the overall arousal ratings were consistent with the nominal arousal of the selected excerpts. However, hues added to video produced no significant effect on arousal ratings, contrary to predictions. This could be due to the domination of the combined effects of other channels of information (e.g., the music and player movement) over the emotional effects of the hypothesized influence of hue on perceived performance (red expected to enhance and blue to reduce arousal of the performance). To our knowledge this is the first study to investigate the impact of these hues upon perceived arousal of music performance, and has implications for musical performers and stage lighting. Further research that investigates reactions during live performance and manipulation of a wider range of lighting hues, saturation and brightness levels, and editing techniques, is recommended to further scrutinize the veracity of the findings.

## Introduction

Music is known to influence emotion in its audience, resulting in common use in many scenarios to manipulate an audience’s mood, such as in shopping, exercise and film (e.g., Cohen, 2010). Furthermore, Huron (2015) has suggested that emotions conveyed by music are enhanced when non-music modalities projected from the same source exhibit emotions that are congruent with the emotion nominally depicted by music. This may be particularly significant in stage lighting. Colored stage lighting regularly accompanies musical performances, from Broadway musicals, to worship bands at mega churches, to sold-out world tours of pop superstars. But what effect does the addition of colored lighting actually have on the emotions conveyed by music to an audience?

The impact of colors on emotion perception have been shown in internet web design (Demir, 2020) and physical spaces (Kurt and Osueke, 2014). Kurt and Osueke (2014) found that spaces with a dominant red color were more likely to be described by as “excitement inducing,” aligning with the view of Birren (1950), who suggested that warmer hues, such as red and yellow, can increase arousal in an individual more than cooler hues, such as blue and green.

In music research Bresin (2005) asked participants to judge how well certain colors fit particular performances of two melodies using rating scales. Overall, red best reflected music expressing anger (high arousal) while blue was most associated with music expressing love (low arousal). Building on this work, Palmer et al. (2013) investigated cross-modal associations between music, color, and emotion. In this study, a significant relationship between all three mediums was observed; suggesting that a cross-modal relationship between color and music was mediated by common emotional associations. For example, “happier” music was associated with a “happy” color (yellow). Musical features also played a role, such as slower music associated with the color blue, because it was mediated by the low arousal emotion of sadness (see also Whiteford et al., 2018; Sugawa et al., 2021). Schubert et al. (2019) asked participants to describe pieces of music with a number of icon-based rating scales, including representations of temperature, color, feelings, and shapes. In this study, slower music was also consistently described as “blue.”

The impact of stage lighting was investigated by Hsiao et al. (2017) who tracked the aesthetic choices made by five professional stage-lighting technicians. They found that the arousal level of the music had a significant relationship with both the choices in saturation and hue made by the technicians. They also found that as the arousal level of the music rose, so did the frequency of red lighting color choice, whilst the opposite result followed the frequency of blue lighting. While the effect of some hues upon arousal are consistent, we have not found research that investigates the more implicit impact of hue upon the emotion expressed by music performance, and yet this is a situation where impact on hue might be having a (currently poorly understood) effect.

The aim of this study was to investigate the effect of hue on an audience’s perception of the level of expression in piano performances. It was hypothesized that (audio/video recorded) performances projected with red hue would be rated higher in arousal than the same performance viewed under a blue hue. To collect a wide range of data and control for possible variables such as habituation and distraction, this study consisted of two experiments: (1) a grouped by color experiment; and (2) a grouped by congruency experiment.

## Experiment 1 (Color Grouping): Method

### Stimuli

Four videos were filmed of four different solo “classical” piano pieces (performed by author JM). Stimulus details and abbreviations for each of these excerpts are presented in Table 1. The nominally high (BeethovenH and ÉlégieH) and low (BachL and PreludeL) arousal pieces were chosen to reflect previous research suggesting a) music generating high arousal is commonly faster, louder and features shorter, more staccato phrases, whilst b) music generating low arousal is commonly slower, softer and features longer, more legato phrases (Bigand et al., 2005; Livingstone and Thompson, 2006). Two sets of the four recordings were prepared by digitally adding a blue hue filter to one set of stimuli and a red hue filter to the other (see ‘Method for coloring videos’ sections in Supplementary Material).

**TABLE 1:** Excerpt details and abbreviations.

Abbreviation	Full title of piece	Composer	Bar Numbers	Duration	Nominal arousal level
BachL/Bach Siloti LOW	“Andante” from the Sonata for Violin Solo in A Minor	Johann Sebastian Bach (Transcribed by Alexander Siloti)	1-11	1 m 2 s	Low
PreludeL/Rach. Prelude LOW	Prelude in D major Op. 23 No. 4	Sergei Rachmaninoff	1-19	1 m 10 s	Low
BeethovenH/Beethoven HIGH	Sonata Op. 27 No. 2 ‘Moonlight’, 3rd Movement	Ludwig Van Beethoven	1-14	0 m 27 s	High
ÉlégieH/Rach. Élégie HIGH	Élégie Op. 3 No. 1	Sergei Rachmaninoff	63-82	0 m 38 s	High

*Two versions of the abbreviations are used, a very short one followed by a slightly longer one, the latter being used to aid in more easily reading graphs of data. All clips commence at the beginning of the work. Clip durations are shown in minutes (m) and seconds (s). Duration of each clip was determined by identifying ostensibly natural cadence points.*

### Participants

The 53 people (41 female, 11 male, 1 non-binary; age range 18–35, M = 21.4, SD = 4.1) participated in Experiment 1. Mean age was 21.4 years, range 18–35, SD = 4.1. Of the 53 participants, 44 played a musical instrument/sang, of which 18 had received lessons for 1-5 years, 8 had received lessons for 6–9 years and 15 had received lessons for 10 or more years. Common instruments played included piano/keyboard (18), voice (11), woodwind (e.g., flute/clarinet/saxophone) (6) and guitar (4). The remaining 9 participants did not play an instrument or sing (that the sum of instrument counts is greater than 53 because some participants reported playing more than one instrument). 26 of the participants listened to the audio from speakers, whilst 27 listened on headphones. All participants reported normal hearing. 33 participants reported watching the videos on a laptop screen, 8 on a computer monitor, 10 on a smartphone and 2 on a tablet.

### Procedure

An online survey was created using the Qualtrics^1^ survey platform. Participants were sorted into two groups at random. The first group viewed all excerpts under a blue filter (blue condition). The second group viewed all the excerpts under a red filter (red condition).

Each group was asked to watch the allocated videos, presented in a randomized order, and to rate the arousal, valence, enjoyment and familiarity of the piece for each excerpt. Arousal was rated on a scale from 0 (“no arousal” e.g., calm) to 10 (“very high arousal” e.g., excited). Valence was rated on a scale from 0 (“negative”) to 10 (“positive”). Enjoyment was rated on a scale from 0 (“did not enjoy”) to 10 (“enjoyed”). Familiarity of the piece was rated on a three point scale: unfamiliar (1), somewhat familiar (2), or very familiar (3).

Once they had completed these questions, participants were asked to provide demographic and computer/audio technical information. The experimental protocol was approved by the University of New South Wales Human Research Ethics Advisory Panel B (approval HC210217).

## Experiment 1: Results and Discussion

**FIGURE 1:**
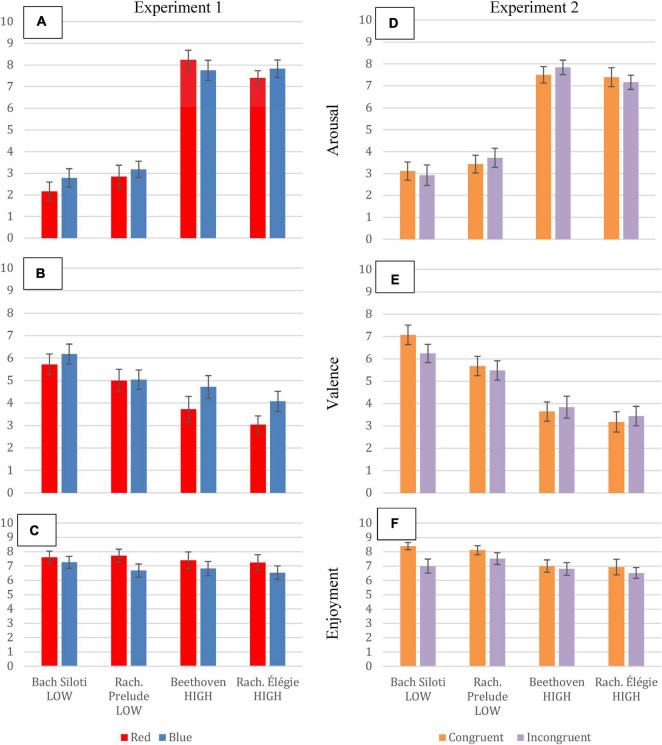
Error bar plots of mean arousal, valence and enjoyment ratings (respectively) in Experiment 1 **(A–C)** and Experiment 2 **(D–F)**. Error bars = ± ±1SE.

Arousal ratings were consistent with the nominal arousal level of all four stimuli, regardless of condition (Figures 1A–C shows the results for valence and enjoyment ratings). Responses were compared for each excerpt between the red condition and the blue condition. As detailed in the Supplementary MaterialTable 2, arousal ratings were statistically identical across condition for each extract. 44 out of 53 participants reported viewing all the videos in full, 9 viewed all at least in part, and none indicated not viewing. As all participants would therefore have glimpsed the red or blue hues at the very least, this should have been enough to trigger a color association, thus strengthening the reliability of arousal ratings. Whilst the lack of difference across the two conditions seemed decisive, the hypothesized change may have been hidden due to participant habituation to the color of the video. Since they viewed each performance with the same coloring, it is possible that the impact of the color on perception of arousal in the music, if indeed there was one, was diminished (Thompson and Spencer, 1966; Rankin et al., 2009). Experiment 2 was therefore designed to validate Experiment 1 by investigating whether color habituation led to the lack of support for the hypothesis.

**TABLE 2:** Mean ratings of familiarity (Experiment 1 and 2).

Experiment 1							
**BachL**		**PreludeL**		**BeethovenH**		**ÉlégieH**	
Red Condition	Blue Condition	Red Condition	Blue Condition	Red Condition	Blue Condition	Red Condition	Blue Condition
1.52	1.57	1.24	1.43	2.08	1.93	1.28	1.43

**Experiment 2**							

**BachL**		**PreludeL**		**BeethovenH**		**ÉlégieH**	
Congruent Condition	Incongruent Condition	Congruent Condition	Incongruent Condition	Congruent Condition	Incongruent Condition	Congruent Condition	Incongruent Condition
1.30	1.44	1.33	1.16	2.04	2.16	1.37	1.20

*Familiarity of the piece was rated on a three-point scale: unfamiliar (1), somewhat familiar (2), or very familiar (3). See Table 1 for abbreviations of the four music excerpts played.*

## Experiment 2 (Congruency Grouping): Method

### Stimuli

The stimuli for Experiment 2 were identical to Experiment 1, with the exception that the participants were shown the videos in different combinations to those of Experiment 1 (see “Procedure”).

### Participants

A total of 53 new participants (34 female, 19 male; age range 18–64, M25.8, *SD* = 10.8 years) were recruited in Experiment 2. This includes one participant who completed the survey twice. Their second response was excluded from the analysis. 45 participants played a musical instrument/sang, of which 18 had received lessons for 1-5 years, 6 for 6-9 years, and 17 for 10 or more years. The remaining 4 participants reported less than 1 year of lessons. Instruments played included 24 piano/keyboard, and 8 participants did not play or sing. 33 of the participants listened to the audio from speakers, whilst 20 listened on headphones. All reported normal hearing. 32 participants reported watching the videos on a laptop screen, 8 on a computer monitor, 10 on a smartphone and 3 on a tablet.

### Procedure

**FIGURE 2:**
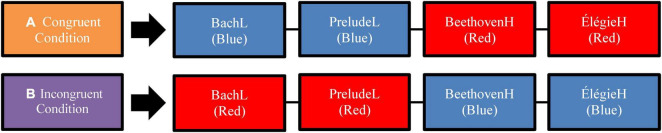
Example of sequences of videos viewed in each condition for Experiment 2. **(A)** Shows an example of the video pipeline in the congruent condition as viewed by participants; **(B)** shows an example of the video pipeline in the incongruent condition as viewed by participants. The actual sequence of the four excerpts (the four boxes) was presented in a different random order for each participant.

Participants undertook a survey identical to that of Experiment 1, except that instead of only viewing the videos under either a red or blue filter, they saw a combination of stimuli with each hue (Figure 2). The participants were again sorted into two groups at random. The first group viewed the excerpts in the “congruent condition” with nominally high arousal excerpts paired with red lighting and low arousal excerpts paired with blue lighting. The second group viewed excerpts in the ‘incongruent condition’: high arousal excerpts with blue light treatment and low arousal excerpts with red light treatment.

## Experiment 2: Results and Discussion

Means were calculated for the ratings of arousal, valence and enjoyment for each condition and are presented in Figures 1D–F (see Supplementary Material Table 3 for additional statistical analyses). Once again, nominal arousal conditions were consistent with participant ratings of arousal for each piece, regardless of condition (Figure 1D). Also evident was that once again, there was no difference in arousal ratings due to condition. The congruent group rated the stimulus BachL as more enjoyable than the incongruent group, and very small trends in the same condition could be observed for all pieces (Figure 1F), which could be explained by color congruence with mood facilitating cognitive fluency (Belke et al., 2010), and hence greater enjoyment. However, neither this result nor the valence ratings were statistically different.

Familiarity ratings (lower half of Table 2) showed little overall difference in ratings between conditions, once again eliminating this variable as a possible confound (in line with the results of Experiment 1 - see top half of Table 2). Participants watched all (46) or some (7) of the videos, with participants who only watched some citing reasons such as lack of time, limited attention, and again, an assumption that they had already understood what they needed to answer the questions, hence listening to a smaller portion of each excerpt. This time, when asked what they thought the study was about, 3 of the 53 participants correctly guessed that the study was testing the effect of colored lighting. A separate analysis was run, excluding the responses of these partcipants. However, there was no significant change to the results. Despite the revised design we still found no evidence to suggest that there was any difference in arousal ratings due to manipulating color. We also ran an analysis for the effect of condition with Experiment 1 and 2 data combined (*n* = 53 per condition). Again, arousal ratings were not significantly higher in the red compared to blue condition (Supplementary Material Table 4). The *a priori* statistical power of this analysis was better than 0.8 (*N* > 102, single-tailed, independent-sample *t*-test).

## General Discussion

The results of this study did not support the hypothesis that hue affects arousal perception of music performance. Several studies used to build our hypothesis measured the level of arousal through direct inspection of color itself (Kurt and Osueke, 2014; Demir, 2020), or based by pairing color and music (Bresin, 2005; Palmer et al., 2013; Schubert et al., 2019). To our knowledge this is the first study that did not intentionally draw participant attention to the presence of colors, rather directing focus to the arousal of the performance. And so perhaps the effect of color, being more implicit, is also small, and the emotions perceived through the auditory channel of the music itself has a far stronger effect.

This apparent dominance of the auditory channel in communicating emotion is also in line with previous studies that explore the relationship between auditory and visual stimuli when communicating emotion. Iop and Pauletto (2021) presented different colored images of doors with a variety of knocking sounds, and found that the aural modality (i.e., the knocking sound) dominated over the visual modality (i.e., the door color) in determining participant perception of emotion. The authors suggested that this was due to the perceived presence of a human at the source of the sound, as opposed to the color of an inanimate object, the door.

Similarly, the emotion in the present study may have been perceived to have come from the human produced sound. With this scenario, the background hue would not be perceived as created by the performer/composer, but rather just another aspect of the environment through which the sound emanates. Non-vocal instrumental musicians can express and convey emotion to a listener, with theories attributing this capacity to empathy and emotional contagion processes because of the resemblance of the music to the characteristics of the human voice (Juslin, 2019) and of shared mental architecture in music and empathy processing (Schubert, 2017). Hence, an empathetic response may dictate the channel that dominates response (whether the musician or the performed music, rather than a background color). This would include multimodal channels of communication, including the body movement of the performer (Broughton and Stevens, 2009). Such an interpretation is still consistent with the ethological signaling theory promoted by Huron (2015): both the signals and cues presented by a performer (i.e., the movements made as the music is performed, including facial expressions) are part of the process of communicating emotion clearly and unambiguously, whereas color manipulation of the video may have had a more ancillary impact.

## Conclusion

This research sought to better understand the effect colored lighting has on the perceived emotional expression of a musical performance, especially relating to the impact of specific hues on arousal. The study did not provide evidence that visual color manipulation of piano performance impacts arousal ratings of the music: that a performance under a red light would be rated higher in arousal than the same performance under a blue light.

As a novel study into color and arousal, there were several limitations. Firstly, the only aspect of color changed within the videos was the hue (red versus blue). It is known that not only hue, but also saturation and brightness can contribute to levels of arousal and valence in emotion (Bresin, 2005; Palmer et al., 2013; Hsiao et al., 2017). Consequently, further research could examine these aspects also, perhaps combining the changing of hue, saturation and brightness within different conditions to see if this enhances the color effect upon emotion when paired with music. Moreover, as it seemed that the effects of the lighting were dominated by other channels of information within the videos, such as the auditory expression of the music and physical expressions of the body of the performer, more stimulating lighting conditions (e.g., mid-performance changes to color, additions of multiple color/pattern combinations, etc.) could enhance these effects.

Another limitation was the online deployment and the consequent variety of different displays used by participants, as data collection was conducted during a “lockdown” due to COVID-19 in Sydney, making data collection in a reasonably well-controlled physical space not possible. It cannot be known from the current study certainty if this variability in screen sizes diminished the impacts of hue on arousal. It is also worth considering the influence of hue in a live performance environment, though this could come with a loss of experimental control because of the challenge of having two identical live performances. A possible solution is through application of immersive virtual environments. For example, Cha et al. (2020) explored participant reactions to color within immersive virtual environments and found a positive relationship between color and arousal, specifically with red as a higher arousal hue. Future research using an immersive virtual environment could better simulate a live environment without the loss of experimental control. Finally, future research could include physiological data collection such as measurement of Motor Corticospinal Excitability or Galvanic Skin Response, to accompany self-report from participants. These physiological measures may have some correlation with mental arousal states (see, e.g., Jola and Grosbras, 2013; Wang et al., 2014).

This study suggests that musicians and stage lighting technicians looking for the best choices in the hue of lighting to enhance the perceived emotional expression of a musical performance should make choices based on artistic discretion, since hue appears to be unlikely to have a significant impact on the overall mood of a music.

## Data Availability Statement

The datasets presented in this article are not readily available because the data collected is confidential. Requests to access the datasets should be directed to ES, e.schubert@unsw.edu.au.

## Ethics Statement

The studies involving human participants were reviewed and approved by UNSW Human Research Ethics Advisory Panel. The patients/participants provided their written informed consent to participate in this study.

## Author Contributions

JM was designed, conducted and documented the project in consultation with all authors. EH and AC edited the additional video and documented. JM, ES, GD, AC, and JZ revised and edited the manuscript. All authors checked all aspects of the design, assisted with the roll out of data, collection and analysis.

## Conflict of Interest

The authors declare that the research was conducted in the absence of any commercial or financial relationships that could be construed as a potential conflict of interest.

## Publisher’s Note

All claims expressed in this article are solely those of the authors and do not necessarily represent those of their affiliated organizations, or those of the publisher, the editors and the reviewers. Any product that may be evaluated in this article, or claim that may be made by its manufacturer, is not guaranteed or endorsed by the publisher.

## Footnotes

1
https://www.qualtrics.com
