# Supplementary material for: Illuminating Music: Impact of Color Hue for Background Lighting on Emotional Arousal in Piano Performance Videos
Source: Front Psychol. 2022 Mar 15;13:828699. doi: 10.3389/fpsyg.2022.828699 (PMC8964794; doi:10.3389/fpsyg.2022.828699)
Supplement: Supplementary file 1 [file Data_Sheet_1.pdf]

Supplementary Material for article

Illuminating music: Impact of color hue for background lighting on emotional arousal in piano performance videos

doi: 10.3389/fpsyg.2022.828699

James McDonald[1], Sergio Canazza[2], Anthony Chmiel[3], Giovanni De Poli[2], Ellouise Houbert[4], Maddalena Murari[2], Antonio Rodà[2], Emery Schubert[1*], J. Diana Zhang[1,5]

[1]Empirical Musicology Laboratory, School of the Arts and Media, University of New South Wales, Sydney, NSW, Australia

[2]Centro di Sonologia Computazionale, Department of Information Engineering, University of Padova, Italy

[3]The MARCS Institute for Brain, Behaviour and Development, Western Sydney University, Sydney, NSW, Australia

[4]Independent researcher, Sydney, Australia

[5]School of Chemistry, University of New South Wales, Sydney, NSW, Australia

*** Correspondence:**
Emery Schubert e.schubert@unsw.edu.au

Contents

1 Method for coloring blue videos 2

2 Method for coloring red videos 4

3 Table 1. *M* and *SE* of ratings for arousal, valence and enjoyment for Experiments 1 and 2. Low and High arousal are denoted with L and H respectively. 6

4 Table 2. Independent Samples *t*-test results for Experiment 1. Low arousal is denoted by L, and High arousal is denoted by H. 7

5 Table 3. Independent Samples *t*-test results for Experiment 2. Low arousal is denoted by L, and High arousal is denoted by H. 8

6 Table 4. Independent Samples *t*-tests for Arousal ratings by piece for effect of hue (Blue vs Red) in Experiments 1 and 2 combined. 9

1 Method for coloring blue videos

The goal was to mimic a blue-tinted spotlight in the scene. The following steps were taken:

1. A single frame was exported from each clip and imported to *Adobe Photoshop 2021*
2. A color balance adjustment layer was added to the frame. As shown below, three properties were manipulated: highlights, midtones, shadows. As the goal was to mimic a blue spotlight, the levels were adjusted so the parts of the image that received the most light (i.e., the highlights) had the most blue adjustment applied, whereas midtones received the second-most blue adjustment, and shadows received the least blue adjustment. The boost to midtones and shadows were adjusted to a point that looked natural (50 and 20, respectively). These values were not pre-conceived; they were selected based on realism.

<table>
<tr>
<td> Highlights: Blue was adjusted to +100. This was done because we are targeting the main source of light in the scene. Adjusting it to be fully blue lets us mimic a blue-tinted singular light source. Red and green were not adjusted, and were left at the default of 0. </td>
<td>
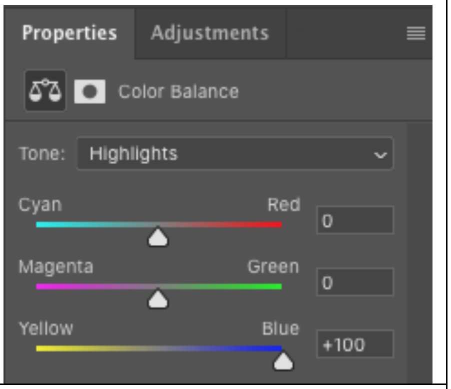

</td>
</tr>
<tr>
<td> Midtones: Blue was adjusted to +50. This level was chosen to simulate a natural light setting; we needed to account for areas that the light touches that would have a blue cast from a blue tinted spotlight, but would not be entirely saturated (i.e., at a level close to 100). Red and green were not adjusted, and were left at the default of 0. </td>
<td>
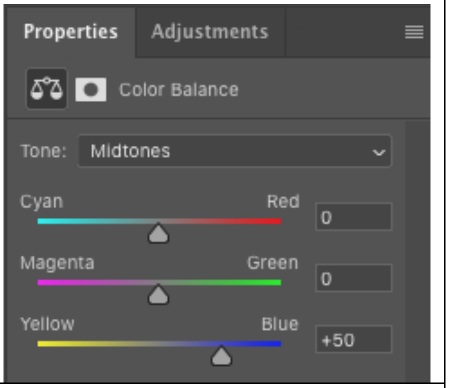

</td>
</tr>
<tr>
<td> Shadows: Blue was to adjusted +20. This gave just a slight hint of blue to the shadows without appearing unnatural. Red and green were not adjusted, and were left at the default of 0. </td>
<td>
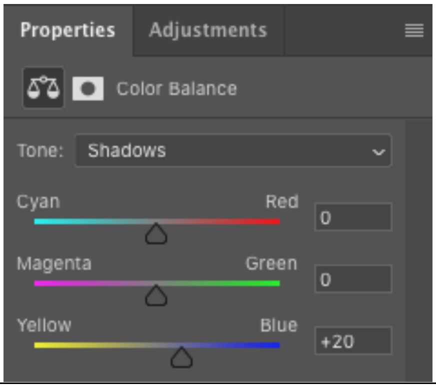

</td>
</tr>
</table>

3. A “curves adjustment” layer was then added on top of the color balance adjustment layer. This was done to further increase the “spotlight” effect on the scene, since that type of lighting is quite severe and dramatic

RGB Shadows were darkened by approximately 5% and highlights were made brighter by approximately 5% (N.B. these values are approximate, as shadow editing is done via visual slider rather than with values).	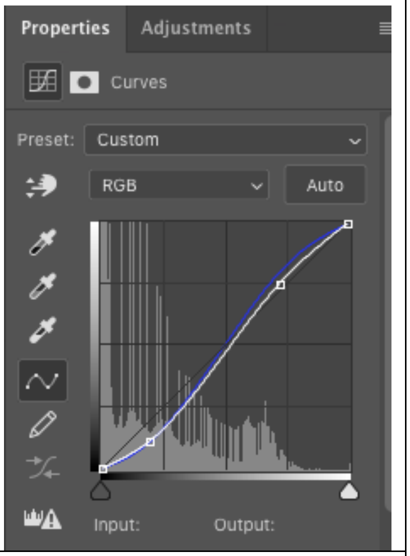
Blue shadows were darkened by approximately 5% and highlights were made brighter by approximately 5%.	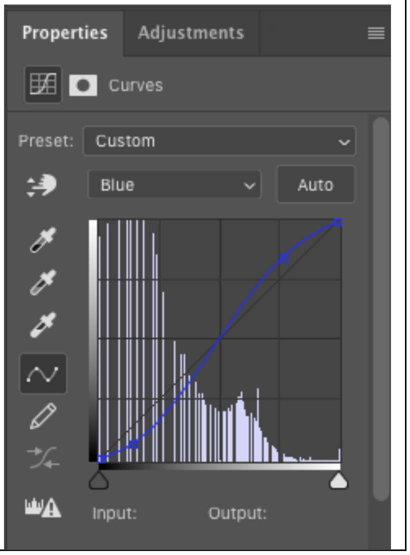

N.B. that Red and Green Curves were not adjusted in step 3

4. This file was then saved as a .psd file, and a LUT (“color lookup table”) was exported from the file, allowing us to apply these color settings uniformly to all clips when editing (Step 6).
5. The videos were edited in *Adobe Premiere Pro 2021*. The LUTs were imported and added to an adjustment layer on top of the cut and trimmed footage.
6. The video was exported as a 1080p .mp4 file.

2 Method for coloring red videos

The goal was to mimic a red-tinted spotlight in the scene. The following steps were taken:

1. The steps were identical as to the blue tint, although as shown below some of the values were different

**Highlights:**<br>Red was adjusted to +70.<br>Blue and green were not adjusted, and were left at the default of 0.	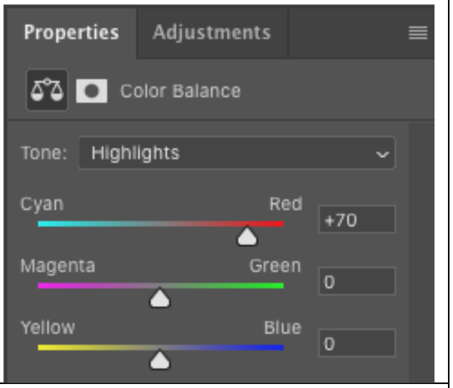
**Midtones:**<br>Red was adjusted to +40.	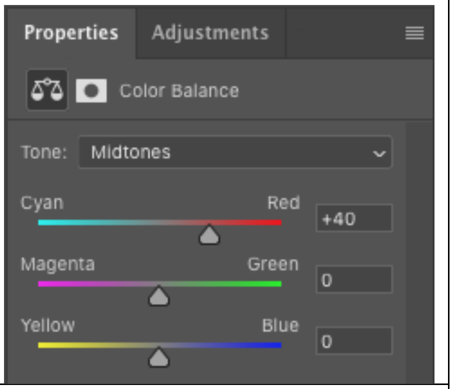
**Shadows:**<br>Red was to adjusted +11.<br>Blue and green were not adjusted and left at the default of 0.	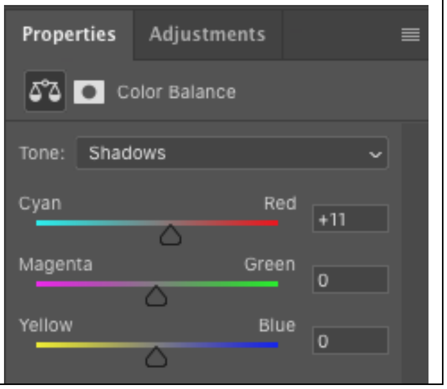

2. As before the “curves adjustment” layer was then added (see Blue section):

RGB Shadows were darkened by approximately 5% and highlights were made brighter by approximately 5%.	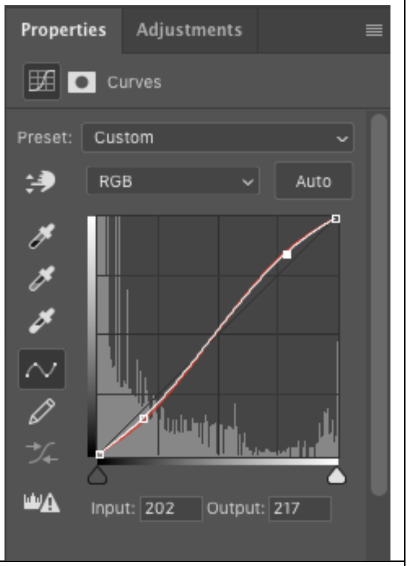
Red shadows were darkened by approximately 5% and highlights were made brighter by approximately 5%.	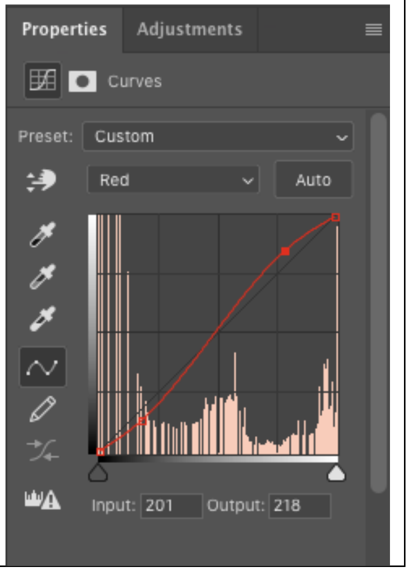

N.B. that the Green and Blue Curves were not adjusted in Step 2.

3. This file was then saved as a .psd file, and a LUT (“color lookup table”) was exported from the file, allowing us to apply these color settings uniformly to all clips when editing (step 5).
4. The videos were edited in *Adobe Premiere Pro 2021*. The LUTs were imported and added to an adjustment layer on top of the cut and trimmed footage.
5. The video was exported as a 1080p .mp4 file.

N.B. All edits were made on a 27 inch *Apple* iMac. To ensure consistency all edits were performed in a single session with consistent lighting conditions.

3 Table 1. *M* and *SE* of ratings for arousal, valence and enjoyment for Experiments 1 and 2. Low and High arousal are denoted with L and H respectively.

	**Experiment 1**											
	**Arousal**				**Valence**				**Enjoyment**			
	Bach L	PreludeL	Beethov H	Élégie H	Bach L	PreludeL	Beethov H	Élégie H	Bach L	PreludeL	Beethov H	Élégie H
Red	2.16	2.84	8.24	7.40	5.72	5.00	3.72	3.04	7.60	7.72	7.40	7.24
Blue	2.79	3.18	7.75	7.82	6.18	5.04	4.71	4.07	7.25	6.68	6.82	6.54
	**Standard Error of the Mean**											
Red	0.43	0.53	0.44	0.33	0.46	0.50	0.57	0.39	0.43	0.45	0.58	0.54
Blue	0.42	0.38	0.46	0.40	0.44	0.44	0.50	0.45	0.42	0.46	0.49	0.47
	**Experiment 2**											
	**Arousal**				**Valence**				**Enjoyment**			
	Siloti L	PreludeL	Beethov H	Élégie H	Siloti L	PreludeL	Beethov H	Élégie H	Siloti L	PreludeL	Beethov H	Élégie H
Congruent	3.11	3.43	7.50	7.39	7.07	5.68	3.64	3.18	8.39	8.11	7.00	6.93
Incongruent	2.92	3.72	7.84	7.16	6.24	5.48	3.84	3.44	7.00	7.52	6.8	6.52
	**Standard Error of the Mean**											
Congruent	0.42	0.40	0.38	0.43	0.43	0.43	0.43	0.45	0.25	0.32	0.43	0.55
Incongruent	0.47	0.44	0.33	0.32	0.41	0.43	0.49	0.43	0.49	0.41	0.44	0.38

Note. Arousal was measured on a scale from 0 (‘no arousal’ e.g., calm) to 10 (‘very high arousal’ e.g., excited). Valence was rated on a scale from 0 (‘negative’) to 10 (‘positive’). Enjoyment was rated on a scales from 0 (‘did not enjoy') to 10 (‘enjoyed’).

4 **Table 2.** Independent Samples *t*-test results for Experiment 1. Low arousal is denoted by L, and High arousal is denoted by H.

Piece	Variable of interest	*p*	*t*
Bach Siloti (L)	Arousal	0.485	1.015
	Valence	0.831	0.702
	Enjoyment	0.903	-0.571
Rachmaninoff Prelude (L)	Arousal	0.252	0.518
	Valence	0.528	0.053
	Enjoyment	0.636	-1.59
Beethoven (H)	Arousal	0.474	-0.747
	Valence	0.439	1.283
	Enjoyment	0.287	-0.753
Rachmaninoff Élégie (H)	Arousal	0.263	0.781
	Valence	0.546	1.69
	Enjoyment	0.729	-0.967

Note. All main effects were non-significant at $p = .05$. For all tests p values are two-tailed, and $df = 52$.

Table 3. Independent Samples *t*-test results for Experiment 2. Low arousal is denoted by L, and High arousal is denoted by H.

Piece	Variable of interest	*p*	*t*
Bach Siloti (L)	Arousal	.944	-.070
	Valence	.107	1.638
	Enjoyment	.023	2.349
Rachmaninoff Prelude (L)	Arousal	.529	-.633
	Valence	.639	.472
	Enjoyment	.348	.948
Beethoven (H)	Arousal	.414	-.824
	Valence	.941	-.075
	Enjoyment	.903	.122
Rachmaninoff Élégie (H)	Arousal	.770	.294
	Valence	.793	-.263
	Enjoyment	.692	.398

Note. All main effects were non-significant at $p = .05$ according to the ANOVA procedure. For all tests p values are two-tailed, and $df = 52$.

6 **Table 4.** Independent Samples *t*-tests for Arousal ratings by piece for effect of hue (Blue vs Red) in Experiments 1 and 2 combined.

Piece	*p*	*t*
Bach Siloti (L)	.361	.917
Rachmaninoff Prelude (L)	.892	-.136
Beethoven (H)	.770	.293
Rachmaninoff Élégie (H)	.958	.053

Note. $n = 53$ for each experiment, meaning overall $N = 106$.
For all tests *p* values are two-tailed, and $df = 104$.
